# Supplementary material for: High association of COVID-19 severity with poor gut health score in Lebanese patients
Source: PLoS One. 2021 Oct 21;16(10):e0258913. doi: 10.1371/journal.pone.0258913 (PMC8530309; doi:10.1371/journal.pone.0258913)
Supplement: S1 Table — (DOCX) [file pone.0258913.s001.docx]

Supplementary Table S1. Detailed description of medical history information.

|  | n | % |
| --- | --- | --- |
| **Chronic diseases** (n=255) |  |  |
| No | 204 | 80.0 |
| Yes | 51 | 20.0 |
| **Type of chronic diseases** (n=51) |  |  |
| Hypertension | 20 | 39.2 |
| Diabetes | 16 | 31.4 |
| Heart Disease | 6 | 11.8 |
| Cholesterol | 6 | 11.8 |
| Liver | 6 | 11.8 |
| Migraine | 5 | 9.8 |
| Allergy | 4 | 7.8 |
| Rheumatism | 3 | 5.9 |
| Other | 3 | 5.9 |
| **Gut problems** (n=255) |  |  |
| No | 197 | 77.3 |
| Yes | 58 | 22.7 |
| **Type of gut problems** (n=58) |  |  |
| Aciduric | 31 | 53.4 |
| Irritable bowel syndrome | 13 | 22.4 |
| Chronic constipation | 10 | 17.2 |
| Ulcer | 8 | 13.8 |
| Chronic diarrhea | 4 | 6.9 |
| **Chronic medication** (n=255) |  |  |
| No | 181 | 71.0 |
| Yes | 74 | 29.0 |
| **Type of chronic medication** (n=74) |  |  |
| Anti-inflammatory | 19 | 25.7 |
| Anti-depression | 5 | 6.8 |
| Other chronic medications | 46 | 62.2 |
