# Supplementary material for: High association of COVID-19 severity with poor gut health score in Lebanese patients
Source: PLoS One. 2021 Oct 21;16(10):e0258913. doi: 10.1371/journal.pone.0258913 (PMC8530309; doi:10.1371/journal.pone.0258913)
Supplement: S2 Table — (DOCX) [file pone.0258913.s002.docx]

Supplementary Table S2. Detailed description of healthy/unhealthy food intake and residual symptoms.

|  | n | % |
| --- | --- | --- |
| ***Unhealthy food intake*** |  |  |
| **Excessive fast-food** **intake** (n=255) |  |  |
| No | 181 | 71.0 |
| Yes | 74 | 29.0 |
| **Excessive sugar intake** (n=255) |  |  |
| No | 159 | 62.4 |
| Yes | 96 | 37.6 |
| ***Healthy food intake*** |  |  |
| **Regular intake of fruits/vegetables** (n=255) |  |  |
| No | 66 | 25.9 |
| Yes | 189 | 74.1 |
| **Regular fermented food intake** (n=255) |  |  |
| No | 25 | 9.8 |
| Yes | 230 | 90.2 |
| **Regular intake of oat/brown bread** (n=255) |  |  |
| No | 154 | 60.4 |
| Yes | 101 | 39.6 |
| **Probiotic intake** (n=255) |  |  |
| No | 247 | 96.9 |
| Yes | 8 | 3.1 |
| ***Residual symptoms*** |  |  |
| **Residual symptoms** (n=193) |  |  |
| No | 141 | 73.1 |
| Yes | 52 | 26.9 |
| **Type of residual symptoms** (n=52) |  |  |
| Fatigue | 23 | 44.2 |
| Anosmia | 11 | 21.2 |
| Cough | 9 | 17.3 |
| Muscle pain | 7 | 13.5 |
| Bone/back pain | 7 | 13.5 |
| Phycological stress | 7 | 13.5 |
| Dysgeusia | 5 | 9.6 |
| Diarrhea | 4 | 7.7 |
| Hearing seeing difficulties | 3 | 5.8 |
| Shortness breath | 3 | 5.8 |
